# An Improved Algorithm to Extract Moiré Fringe Phase for Wafer-Mask Alignment in Nanoimprint Lithography

**DOI:** 10.3390/mi15121408

**Published:** 2024-11-22

**Authors:** Feifan Xu, Yinye Ding, Wenhao Chen, Haojie Xia

**Affiliations:** 1 Anhui Province Key Laboratory of Measuring Theory and Precision Instrument, Hefei University of Technology, Hefei 230009, China; 2021110003@mail.hfut.edu.cn; 2 Anhui Provincial Engineering Research Center of Semiconductor Inspection Technology and Instrument, Hefei University of Technology, Hefei 230009, China; dingyy@mail.hfut.edu.cn; 3 School of Instrument Science and Opto-Electronics Engineering, Hefei University of Technology, Hefei 230009, China; 2021170036@mail.hfut.edu.cn

**Keywords:** lithography alignment, moiré fringe technology, phase extraction algorithm, nanoimprint lithography, misalignment measurement

## Abstract

This paper proposes an improved algorithm based on the phase extraction of the Moiré fringe for wafer-mask alignment in nanoimprint lithography. The algorithm combines the strengths of the two-dimensional fast Fourier transform (2D-FFT) and two-dimensional window Fourier filtering (2D-WFF) to quickly and accurately extract the fundamental frequencies of interest, eliminate noise in the fundamental frequency band by using the threshold of the local spectrum, and effectively suppress spectral leakage by using a Gaussian window with outstanding sidelobe characteristics while overcoming their limitations, such as avoiding the time-consuming parameter adjustment. The phase extraction accuracy determines the misalignment measurement accuracy, and the alignment accuracy is enhanced to the nanometer level, which is 15.8% and 6.6% higher than 2D-FFT and 2D-WFF, respectively. The results of simulations and experiments confirm the feasibility and rationality of the algorithm.

## 1. Introduction

Nanolithography is a critical technology that is extensively used in integrated circuit manufacturing [1,2]. However, with the development of lithography, the technology nodes have gradually approached the physical limit of Moore’s law [3]. To overcome this limitation, nanoimprint lithography (NIL) has risen as a highly promising technology and is being widely used in fields such as augmented reality, three-dimensional (3D) microsensors, and biological nanostructures. NIL is based on the principle of mechanical replication, and the transfer of lithographic patterns is realized by contact imprinting. Compared with traditional lithography technology [4], NIL can overcome the challenges associated with the optical diffraction limit and is characterized by high resolution, high precision, and low cost. However, to achieve effective NIL, the higher accuracy of the overlay alignment must be ensured. Typically, the alignment accuracy must be 15% to 20% of the minimum feature size [5], and thus, future NIL must exhibit nanometer-level alignment accuracy.

With active research, several lithography alignment techniques have been developed, with geometric imaging [6], diffracted light intensity [7], linear zone plate [8], and Moiré fringe alignments [9,10,11,12,13,14] being representative. Although these techniques exhibit high accuracy and sensitivity, the first three techniques are susceptible to the variation in the wafer-mask gap and thus are unsuitable for contact NIL. In comparison, Moiré fringe alignment is robust against the variation in the gap between two alignment marks [15]. NIL alignment based on the Moiré fringe has been extensively studied [16,17,18].

In lithography misalignment measurement, line-grating alignment marks are extensively used to produce single-frequency Moiré fringes. These fringes consist of three main spectrum components: the zero frequency, denoting the background intensity; and the +1 and −1 level fundamental frequencies, which carry the phase information [19]. Misalignment can be obtained and adjusted by extracting the phase difference from the Moiré fringes. Therefore, the misalignment measurement accuracy strongly depends on the phase extraction accuracy. The two-dimensional fast Fourier transform (2D-FFT), as a global processing algorithm, is extensively employed for extracting the phase information of fringe patterns [20]. However, owing to the fundamental frequency component typically exhibiting a wide frequency band, this method may not effectively filter out the noise within that band. The noise can cause phase distortion, thereby deteriorating the phase extraction accuracy. Furthermore, the effect of discretized sampling by the camera, asynchronous sampling of algorithms, and non-integer multiples fringe period truncation may lead to spectral leakage [9], resulting in a degradation of the phase extraction accuracy. To address these challenges, the two-dimensional window Fourier transform (2D-WFT) has emerged as a popular method [21,22], which uses a Gaussian window with outstanding sidelobe characteristics to locally truncate and weight the fringe image, thereby reducing spectral leakage. Additionally, 2D window Fourier filtering (2D-WFF) [23], as a derivative algorithm of 2D-WFT, uses the threshold of the local spectrum to effectively suppress noise. However, when processing fringes, 2D-WFF must adjust the optimal parameters (nine parameters such as window size and position, sampling interval, and threshold size) within each small window to achieve optimal spectrum selection and filtering, which is time-consuming and hinders real-time processing.

To address these problems, we propose an accurate and fast improved phase extraction algorithm based on 2D-FFT and 2D-WFF, named the 2D Fourier window transform (2D-FWT). The proposed algorithm combines the strengths of 2D-FFT and 2D-WFF to effectively suppress noise and spectral leakage while avoiding time-consuming parameter adjustment. Specifically, the 2D-FWT algorithm exploits the fast computation and frequency resolution abilities of 2D-FFT in extracting the fundamental frequency. Subsequently, the local truncation and window weighting strategy of 2D-WFF is used to eliminate noise disturbances and suppress spectral leakage. Simulation and experimental results confirm the feasibility and effectiveness of the 2D-FWT algorithm. Compared with both classical algorithms, 2D-FWT enables high-precision phase extraction, thereby achieving real-time (0.26 s) nanoscale lithography alignment measurements.

The remainder of this paper is outlined as follows. The alignment principle, 2D-FWT and unwrapping algorithms, and annotations of these algorithms are described in Section 2. Section 3 describes the simulation-based evaluation of the performances of different algorithms. Section 4 presents details of the experiments and discusses the results. Section 5 presents the concluding remarks.

## 2. Principle

### 2.1. Alignment Principle

**Figure 1:**
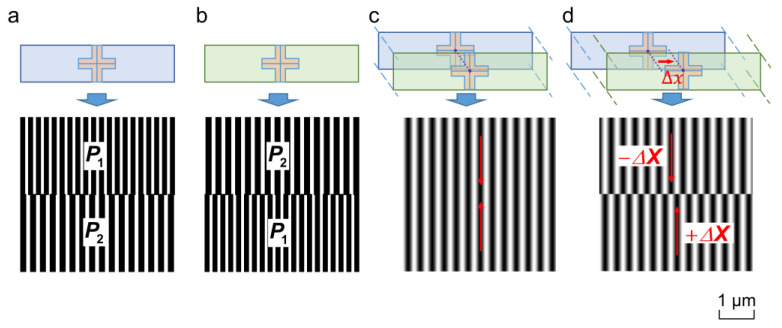
Alignment marks and Moiré fringe. (**a**) Wafer and (**b**) mask marks. (**c**) Perfectly aligned fringe (**d**) and misaligned fringe.

Figure 1a,b shows two sets of alignment marks, each of which consists of upper and lower double gratings featuring marginally distinct periods ($P_{2}\approx 1.1P_{1}$). In the alignment process, any small misalignment between the wafer and the mask is amplified by Moiré fringes, and the upper and lower fringes are displaced along opposite directions. Figure 1c,d shows the Moiré fringe patterns when the wafer and mask are perfectly aligned and misaligned, respectively. Generally, the fringe pattern intensity in the upper and lower parts of Figure 1c,d can be represented as

(1) $$I_{up}\left(x,y\right)=a\left(x,y\right)+b\left(x,y\right)\cos\left[2\pi \left(f_{1}-f_{2}\right)x+2\pi f_{1}∆x\right],$$

(2) $$I_{down}\left(x,y\right)=a\left(x,y\right)+b\left(x,y\right)\cos\left[2\pi \left(f_{1}-f_{2}\right)x-2\pi f_{2}∆x\right],$$

where $I\left(x,y\right),\;a\left(x,y\right)$, and b(x,y) denote the fringe recording intensity, background intensity, and contrast between light and dark fringes, respectively. $f_{1}=1/P_{1}\;and\;f_{2}=1/P_{2}$ denote the spatial frequency of both gratings. Both sets of fringes include the term $2\pi \left(f_{1}-f_{2}\right)x$ in their spatial frequencies, which means that both exhibit the same periodicity and can be regarded as carrier fringes with a carrier frequency of $\left(f_{1}-f_{2}\right)$. Note that ∆x  and ∆X represent the actual misalignment and the Moiré fringe displacement along the *x*-axis, respectively. The relationship between them is $∆X=\left[{(P}_{1}+P_{2}\right)/\left|P_{2}-P_{1}\right|]*∆x$. The phases of the misalignment fringe patterns are offset, and this phase difference can be obtained using the phase extraction algorithm

(3) $$∆\varphi \left(x,y\right)=\varphi_{up}\left(x,y\right)-\varphi_{down}\left(x,y\right)=2\pi (f_{1}+f_{2})∆x,$$

where $\varphi_{up}\left(x,y\right)=2\pi \left(f_{1}-f_{2}\right)x+2\pi f_{1}∆x$ and $\varphi_{down}\left(x,y\right)=2\pi \left(f_{1}-f_{2}\right)x-2\pi f_{2}∆x$ are the spatial phases of the upper and lower fringes, respectively. Then, the misalignment can be calculated as follows:

(4) $$∆x=\frac{∆\varphi \left(x,y\right)}{2\pi \left(f_{1}+f_{2}\right)}.$$

The misalignment measurement accuracy depends on the phase extraction accuracy and the alignment mark fabrication accuracy. In general, the latter is challenging to improve in the later stages of the process because it is limited by the fabrication process. Therefore, the accuracy of the phase extraction algorithm must be enhanced to accurately measure the phase difference.

The following sections describe the principles of the 2D-FFT, 2D-WFF, and 2D-FWT phase extraction algorithms.

### 2.2. Phase-Processing Algorithm

#### 2.2.1. Improved 2D-FWT Algorithm Theoretical Model

The 2D-FFT and 2D-WFF algorithms are typically used for phase extraction of interferometric fringes by spatial-to-frequency conversion. Details regarding these algorithms can be found in Ref. [24]. To precisely measure the misalignment and overcome the limitations of these algorithms, we propose an improved algorithm named 2D-FWT, and its principle is described in the following text.

First, the fringe is processed by 2D-FFT. A 2D Hanning bandpass filter with superior sidelobe performance [25] is used to eliminate the zero frequency, reduce noise near the fundamental frequency, and extract the +1 level spectrum. The spectrum is then subjected to the inverse Fourier transform (IFT):

(5) $$\bar{I}\left(x,y\right)=F^{-1}\left\{F{(H}_{+1}\left[I\left(x,y\right)\right])\right\},$$

where $F^{-1}$ and F represent the IFT and FT operators, respectively. $H_{+1}$ denotes the Hanning bandpass filter, which is centered at the peak of the fundamental frequency. $\bar{I}\left(x,y\right)$ denotes the spatial distribution of the spectrum that excludes the other disturbance terms and contains only the target phase information. $\bar{I}\left(x,y\right)$ is used as the input signal for 2D-WFF processing.

(6) $$Sf\left(\mu ,\nu ,\xi ,\eta \right)=\int_{-\infty }^{\infty }\int_{-\infty }^{\infty }\bar{I}\left(x,y\right)g_{\mu ,\nu ,\xi ,\eta }^{*}\left(x,y\right)dxdy,$$

where $g_{\mu ,\nu ,\xi ,\eta }\left(x,y\right)=g\left(x-\mu ,y-\nu \right)\exp\left(j\xi x+j\eta y\right)$ and $g_{\mu ,\nu ,\xi ,\eta }^{*}$ is its complex conjugate, defined as the WFT kernel. Parameters μ and ν denote the shift factor of the window center in the x and y directions, respectively. As μ and ν increase, the window moves forward to cover the whole domain, and the corresponding frequency information in the frequency domain is ξ and η, respectively. The $g\left(x,y\right)$ expresses the normalized Gaussian window function, whose symmetry and smoothness contribute to a more concentrated wave function shape, conforming with the principle of minimum Heisenberg Uncertainty [26]. The Gaussian window function helps optimize the trade-off between the time and frequency resolution, and it is expressed as follows.

(7) $$g\left(x,y\right)=\frac{1}{\sqrt{\pi \sigma_{x}\sigma_{y}}}\exp\left(-\frac{x^{2}}{2\sigma_{x}^{2}}-\frac{y^{2}}{2\sigma_{y}^{2}}\right),$$

where $\sigma_{x}\;$ and $\sigma_{y}$ denote the standard deviation (SD) of the Gaussian function in the x and y directions, respectively, to control the spatial expansion. Theoretical and experimental investigations have demonstrated that 2D-WFF can effectively suppress noise in fringe patterns, with a signal-to-noise ratio gain of approximately 20 [27]. In the WFF process, the noise spectrum is filtered by setting a threshold (*thr*) as follows.

(8) $$\bar{Sf}\left(\mu ,\nu ,\xi ,\eta \right)=\left\{\begin{array}{c} Sf\left(\mu ,\nu ,\xi ,\eta \right)\;\;\;\;\;\;if\left|Sf\left(\mu ,\nu ,\xi ,\eta \right)\right|\geq thr,\\ 0\;\;\;\;\;\;\;\;\;\;\;\;\;\;\;\;\;\;\;\;\;\;\;\;\;\;\;if\left|Sf\left(\mu ,\nu ,\xi ,\eta \right)\right|<thr, \end{array}\right.$$

where $\bar{Sf}\left(\mu ,\nu ,\xi ,\eta \right)$ is the filtered window Fourier spectrum. Finally, it is converted to the spatial domain to reconstruct the high-quality interference fringes as follows:

(9) $$\tilde{I}\left(x,y\right)=\frac{1}{4\pi^{2}}\int_{-\infty }^{\infty }\int_{-\infty }^{\infty }\int_{-\infty }^{\infty }\int_{-\infty }^{\infty }\bar{Sf}\left(\mu ,\nu ,\xi ,\eta \right)g_{\mu ,\nu ,\xi ,\eta }\left(x,y\right)d\xi d\eta d\mu d\nu ,$$

The phase of the fringe is defined as

(10) $$\varphi \left(x,y\right)={tan}^{-1}\left[\frac{Im\left[\tilde{I}\left(x,y\right)\right]}{Re\left[\bar{\tilde{I}}\left(x,y\right)\right]}\right].$$

where Im[] denote the imaginary parts of $\tilde{I}\left(x,y\right)$, and Re[] denote the real parts of $\tilde{I}\left(x,y\right)$. The $\varphi \left(x,y\right)$ is distributed within the range (−π,π) and must undergo unwrapping to acquire a continuous phase. The unwrapping theory of phase is discussed briefly in Section 2.2.3 below.

#### 2.2.2. Annotations for Implementations of Algorithms

Considered algorithms can be flexibly used in practical applications; here, we clarify their implementation details to ensure the reproducibility of the results.

(1) The conventional strategy for 2D-FFT implementation is used. A 2D Hanning bandpass filter is used to extract the +1-level spectrum. The filter bandwidth is manually determined based on the spectrum distribution characteristics. Minimize the filter size to reduce noise interference when using 2D-FFT, and maximize the size to extract the complete spectrum information using the 2D-FWT. Thus, there is a trade-off between noise or spectrum loss and spectrum extraction accuracy.
(2) To balance the frequency and time resolutions, the size of the Gaussian window function is set as $\left[{4\sigma }_{x}+1]\times [4\sigma_{y}+1\right]$, with $\sigma_{x}=\sigma_{y}=10\;\mu m$. To reduce spectral leakage, the frequency range must be extended to $\left[3/\sigma_{x},3/\sigma_{y}\right]$. Given that the frequency range of the fringe pattern is estimated by the FT as $\left[\xi ,\eta \right]=[a,b]\times [c,d]$, the spectral range of the FWT is set as $\left[\xi ,\eta \right]=[a-3/\sigma_{x},b+3/\sigma_{x}]\times [c-3/\sigma_{y},d+3/\sigma_{y}]$ [28]. The sampling frequency $\left[\xi_{i},\eta_{i}\right]$ must be adequately small to accurately locate the local frequencies in the frequency range. However, to ensure a reasonable run time, the sampling interval only needs to cover the window uniformly for dense sampling. Therefore, the sampling frequency can be set as $\left[\xi_{i},\eta_{i}\right]\in [1/\sigma_{x},1/\sigma_{y}]$, and a finer sampling frequency can be set if higher precision sampling is desired. In addition, the choice of the threshold considerably affects the phase extraction results: an excessively large threshold may lead to distortion of the eigen-signal, and an excessively small threshold may lead to noise leakage. Both cases may result in phase distortions. A larger threshold interval can be selected to achieve nearly optimal filtering results, followed by fine-tuning based on the desired phase extraction effect.
(3) When processing fringe patterns with the WFF, the lower frequency limit $c-3/\sigma_{y}$ of the right half-plane of the spectral range must be located between the 0- and +1-level spectrum to completely extract the spectrum and avoid the infiltration of the 0-level spectrum. In this context, the selection of the lower frequency limit is critical for algorithm debugging and must be undertaken as the first step. However, the small window size results in a very small distance between both spectrums, and as such, an adjustment is very time-consuming. After setting the lower frequency limit, the threshold can be further adjusted to optimize the results. Fortunately, the proposed 2D-FWT does not need to consider this.

#### 2.2.3. Phase Unwrapping Algorithm

Phase unwrapping is critical to ensure real-time processing and precisely measure misalignment. Therefore, we introduce the weighted least squares (WLS) phase unwrapping algorithm [29], which treats phase discontinuities by introducing weighting coefficients. The weighting coefficients are determined based on factors such as the noise level and signal intensity, and they are used to limit the effects of the residuals and noise. WLS employs an iterative formulation based on Poisson’s equation to control the propagation of smoothing errors and mitigate the error propagation from low-quality points. By appropriately setting the weight coefficients, WLS can optimize the objective function and ensure robust phase unwrapping.

## 3. Simulation

To confirm the robustness of the 2D-FWT, a simulated fringe pattern was performed using MATLAB R2022b. We introduced a red source with a wavelength of 625 nm. The objective magnification was 8, and the CMOS pixel array had dimensions of 1280 × 1024 with a pixel size of 5.2 μm. The line widths of the gratings (Figure 1a,b) were $P_{1}\;4=\mu m$ and $P_{2}=4.4\;\mu m$, whose aperture-to-grating ratio was 1/2, and the size was 512 μm×512 μm. The period of Moiré fringes (Figure 1c,d) was $P_{m}=P_{1}P_{2}/\left|P_{2}-P_{1}\right|=44\;\mu m$.

### 3.1. Pre-Processing

**Figure 2:**
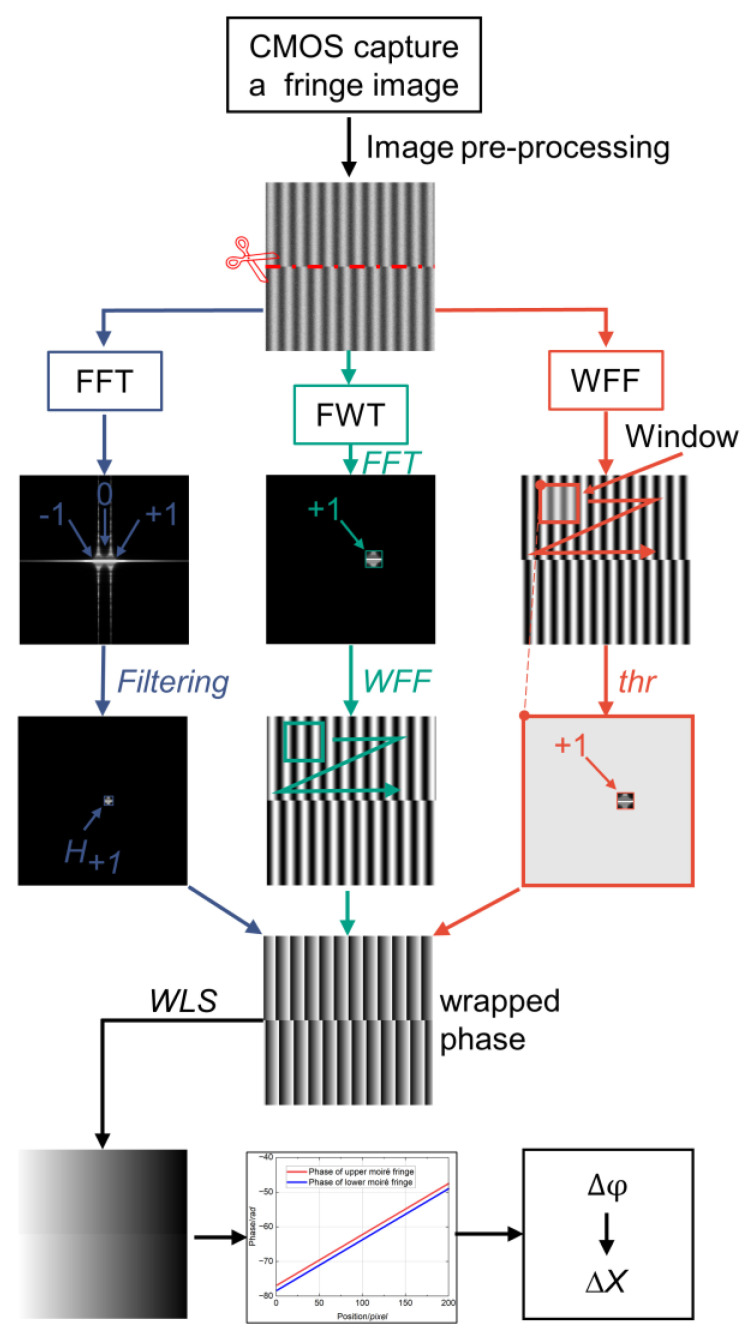
Process flow to extract the fringe phase.

The complete phase extraction process is illustrated in Figure 2. The key considerations can be summarized as follows.

(1) Image pre-processing: The fringe contrast may be reduced due to illumination disturbances and mark fabrication defects. Morphological methods can improve the contrast. If the fringe is severely disturbed by noise, spatial filtering can be applied for noise reduction without losing the critical information of the original image.
(2) Image segmentation: Because of the truncation of the Moiré fringe along the middle misalignment, the phase is discontinuous at this position. To solve this problem, the image is segmented into upper and lower parts along the middle pixel mutation and processed separately before performing phase extraction. This step can avoid the loss of key information of the fundamental frequency and reduce phase error.
(3) Integral multiple truncations: According to the sampling theorem, the captured Moiré fringe pattern is truncated using an integer period (${N\times P}_{m}$) to reduce spectral leakage and improve the accuracy of the FWT algorithm.

After determining the unwrapping phase, two sets of 1D signals are obtained by averaging along the direction perpendicular to the fringe arrangement. This approach can effectively filter out part of the image noise and avoid local distortion.

### 3.2. Robustness Testing of Algorithms

**Figure 3:**
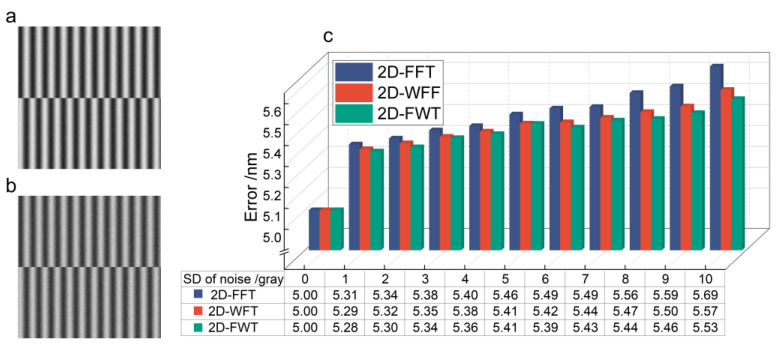
Results of comparative analysis in noisy conditions: fringe pattern with the noise of (**a**) SD = 5 *gray level* (**b**) SD = 10 *gray level*. (**c**) The absolute error of misalignment values.

To confirm the accuracy and robustness of the 2D-FWT algorithm, we preset the misalignment between the wafer and mask as ∆x=0.5 μm. To assess the performance under different noise levels, additive random noise with SD ranging from 1 *gray level* to 10 *gray level* was added to an interference fringe image with gray levels from 0 to 255 levels, and 10 simulation runs, with the noise regenerated randomly at the beginning of each run. The noise fringe with SDs of 5 *gray level* and 10 *gray level* are shown in Figure 3a,b, respectively. Furthermore, a random error of 5 nm was introduced to represent the mark fabrication defects error and PZT movement error. The algorithm parameters were selected as discussed in Section 2.2.2. The errors of the obtained misalignment measurement values are shown in Figure 3c. The 2D-FFT, as a global algorithm, is susceptible to noise, which adversely affects the phase extraction accuracy. In contrast, both 2D-FWT and 2D-WFF use threshold and Gaussian windows to effectively mitigate noise and spectral leakage. The 2D-FWT technique can accurately extract the fundamental frequencies using 2D-FFT while filtering out the zero-frequency components and other interferences. In the subsequent processing of the +1-level fringe pattern using 2D-WFF, the disturbance of the zero and fundamental frequencies does not need to be considered, which helps to reduce the time required for parameter adjustment. The slight improvement in the accuracy is attributable to suppressing interference from nearby zero frequency and further reduction in spectrum loss within each small window. In this manner, nanometer-level alignment accuracy can be attained. To investigate the error sources, the alignment process is iterated, excluding PZT movement and mark fabrication errors from consideration. Under these ideal conditions, the theoretical alignment precision achieves sub-nanometer levels, as listed in Table 1.

## 4. Experiment

### 4.1. Alignment Marks Fabrication

**Figure 4:**
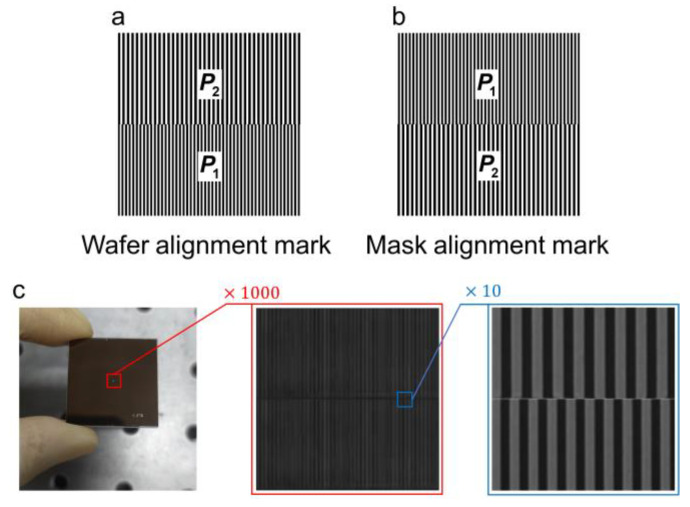
Designed alignment marks. (**a**) Wafer and (**b**) mask marks designed with L-edit. (**c**) Marks fabricated through EBDW.

These alignment marks are designed by Electronic Design Automation (EDA) commercial software (Tanner, L-edit, version 16.30), as shown in Figure 4a,b and fabricated through electron beam direct writing (EBDW) lithography. The local microscopic images are shown in Figure 4c. The grating periods are $P_{1}=4\;\mu m$ and $P_{2}=4.4\;\mu m$, and the size is 0.5 mm×0.5 mm. While the alignment experiment is conducted solely in the 1D *x*-direction, the attainment of 2D measurements becomes feasible by incorporating two sets of orthogonal alignment marks in the *x*- and *y*-directions.

### 4.2. Measurement System

**Figure 5:**
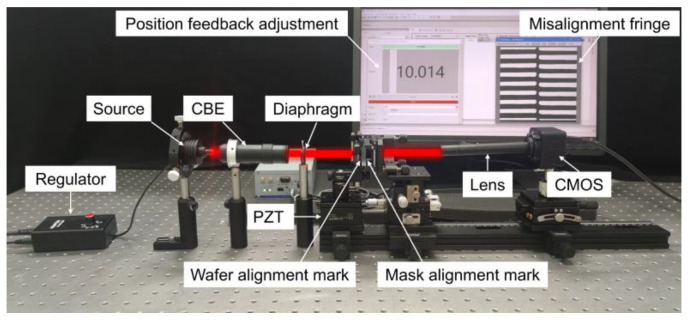
Experimental validation system.

The NIL alignment validation system is shown in Figure 5. A red LED light source (620~630 nm, 5 mW output power) is collimated by a collimated beam-expanding (CBE) system and stray light is filtered using a diaphragm. The LED effectively avoids interference with Moiré fringes caused by scattering. The wafer mark is located on a piezoelectric transducer (PZT, P-612.ZSL, range =100 μm, resolution = 0.2 nm, Physik Instrumente, Karlsruher, Germany) and the mask mark is affixed to a manual XYZ stage (100 mm travel, resolution = 1 μm). Some symmetric diffraction levels of these marks placed in the Talbot light field encounter each other and interfere to produce Moiré fringe. To reduce image distortion, the modulated Moiré fringe is imaged using an MML8-ST110D (8×, MORITEX, Yokohama, Japan) and a CMOS camera (CGU2-130 M, Cgimagetech, Hangzhou, China). The captured patterns are processed and analyzed, and the measurement results are fed back to the PZT for closed-loop adjustment by the feedback adjustment system. 

Before the experiment, the misalignment needs to be zeroed out as much as possible to generate a visually perfectly aligned Moiré fringe pattern, ensuring that there is enough distance in the step experiment to avoid the fringes repeating themselves. The complete experiment was performed on an optical platform in an ultra-clean and vibration-isolation laboratory to reduce external vibrations and disturbances. To ensure the sinusoidal characteristic of Moiré fringes, we meticulously controlled the optical path in our experiments, including the light source, the lens, and the relative angle between the alignment marks. This helped to ensure that the incident light formed a sinusoidal wave between the wafer and the mask grating marks. High-quality optical components (LED and collimated beam expanding lens) are used to ensure the quality and collimation of the light, thus reducing the possibility of non-sinusoidal interference.

### 4.3. Experimental Results

**Figure 6:**
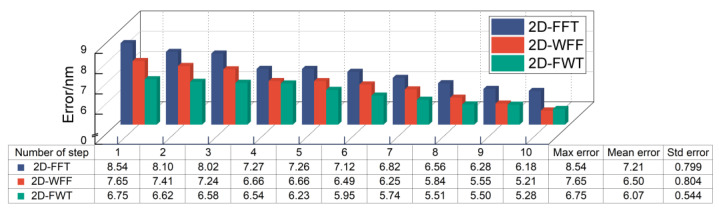
Maximum error (Max error), mean error (Mean error), and standard deviation of the error (Std error) of different algorithms in 10 nm steps.

To confirm the accuracy of the proposed algorithm, we perform 10 repetitive step experiments in the *x*-direction with the minimal step distance of 10 nm (the experiment is in no order). Before the experiment, we processed the captured fringe as described in Section 3.1 and the result is shown in Figure 6. The maximum error, mean error, and standard deviation of error associated with the proposed 2D-FWT are 6.75 nm, 6.07 nm, and 0.544 nm, respectively. Compared with the classical 2D-FFT and 2D-WFF, the alignment accuracy of the 2D-FWT is 15.8% and 6.6% higher, respectively. Furthermore, the optimal parameter adjustment time is greatly reduced when the 2D-FWT is used to process the experimentally acquired fringe. The runtime for a single instance is limited to 0.26 s (averaged over 10 runs and calculated by a software timer). In other words, 2D-FWT can achieve real-time nanoscale misalignment measurements.

**Figure 7:**
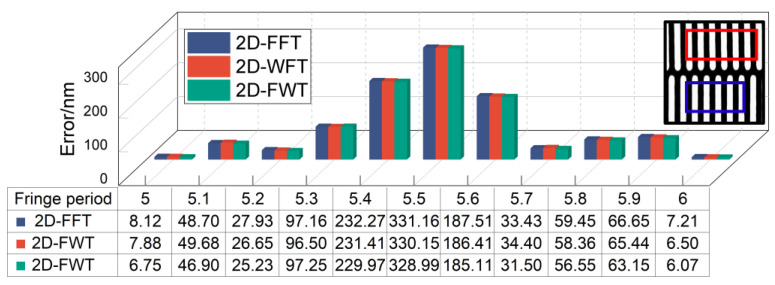
Alignment error when the fringe truncating length is 5 to 6 periods.

To clarify the effect of the fringe period truncation on the alignment accuracy, experiments were conducted with a fringe of different truncating periods, and the result is shown in Figure 7. When the truncate length is an integer period, the alignment accuracy obtained by all three algorithms is on the nanometer level. In contrast, the alignment error is up to approximately 330 nm. Thus, the truncated length of the fringe has a significant effect on the alignment accuracy. This phenomenon can be explained by spectral leakage, which is related to the sidelobe behaviors of the window. A window function with a lower peak sidelobe level and higher sidelobe decaying rate is introduced to alleviate the effect of spectral leakage, thereby decreasing the alignment error. The signal length also greatly influences the measurement accuracy, with a signal of period 6 being significantly more accurate than a signal of period 5. This is because increasing the signal length improves the spectral resolution and can more accurately represent the spectral characteristics of the fringe signal, further reducing spectral leakage.

**Figure 8:**
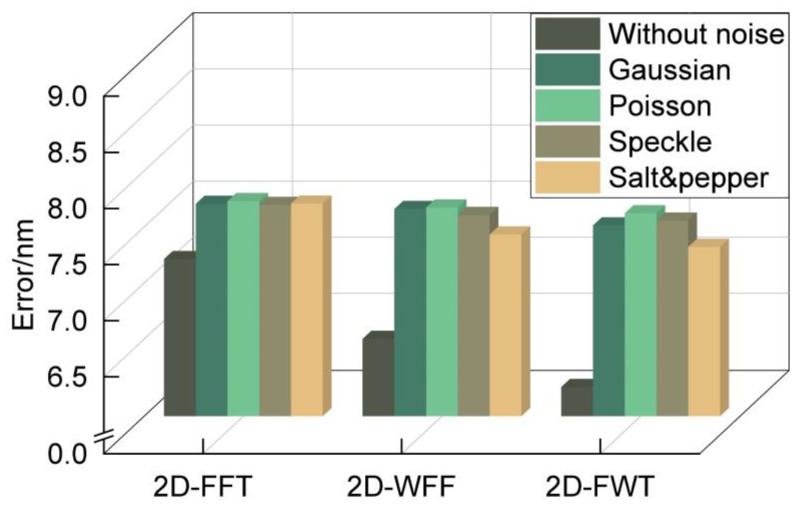
Robustness experiments under different types of noise.

The simulation results (Section 3.2 demonstrate the robustness of the algorithm under the same additive random noise with different SDs. However, the robustness of the algorithm against various types of noise must be evaluated. To this end, we applied MATLAB post-processing techniques to add different types of noise to the captured Moiré fringe pattern, including Gaussian white noise (with a mean of 0 and variance of 0.02), Poisson noise, speckle noise (with a variance of 0.02), and salt and pepper noise (with a noise density of 0.02). The results in Figure 8 demonstrate that the proposed algorithm could still achieve nanometer-level alignment accuracy even under different background noise. In other words, the 2D-FWT has good anti-noise capability for all types of noise.

**Figure 9:**
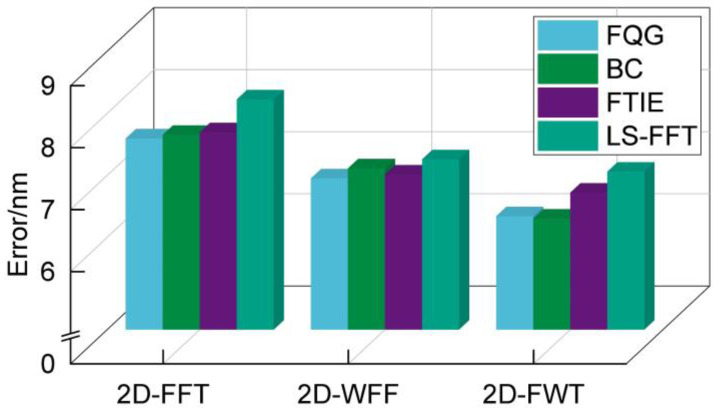
Absolute errors of alignment are obtained by different types of algorithms.

Phase extraction algorithms typically need to be used in combination with unwrapping algorithms in practice [22]. Therefore, we selected several classical unwrapping algorithms to verify the universality of 2D-FWT. In the path-following category, two algorithms were chosen: the fast quality-guided (FQG) algorithm [30] and the Goldstein branch-and-cut (BC) algorithm [31]. These algorithms are known for their effectiveness in resolving phase discontinuities and producing accurate unwrapped phase maps. In the path-independent category, two algorithms are chosen: the fast transport of intensity equation (FTIE) algorithm [32] and the FFT-based least squares (LS-FFT) algorithm [33]. These algorithms are known for their efficiency and robustness in phase unwrapping. The absolute errors of the result are shown in Figure 9. All the considered unwrapping algorithms achieved nanometer-level alignment accuracy. Notably, the algorithms in the path-following category were more accurate than those in the path-independent category. However, path-following algorithms require more computational time owing to their iterative nature and the need to handle complex phase discontinuities. Thus, the choice of the unwrapping algorithm depends on the application requirements and the desired balance between accuracy and computational efficiency.

To evaluate the sensitivity, another experiment is conducted using two other marks with grating periods of T1 = 6 μm and T2 = 8 μm and a modulated Moiré fringe period of $T_{m}=24\;\mu m$. The algorithm parameters, experimental data collection methods, and evaluation criteria were the same as those in the previous experiments to ensure consistency and universality. The results are summarized in Table 2. The alignment accuracy obtained by all three algorithms decreases in comparison with the original experiment. In general, the measurement sensitivity is higher when grating alignment marks with smaller periods are used. Therefore, the use of grating alignment marks with smaller periods prepared using more advanced grating etching tools can help enhance the alignment accuracy. However, this does not mean that a smaller grating size is better. If the dimensions of the grating structure approach or fall below the wavelength of the incident light, the conventional scalar diffraction theory becomes inadequate, necessitating the use of finite-difference time-domain [34] or finite-element method [35] for analysis. Additionally, scalar propagation [36] (such as Fresnel/Fraunhofer approximations or Rayleigh-Sommerfeld) and vector propagation [37] algorithms hold potential advantages in accurately simulating the propagation of an optical field. These algorithms promise enhanced phase recovery and alignment accuracy. Future research could investigate the application of these techniques to improve alignment accuracy, while considering the trade-off between computational complexity and real-time requirements.

### 4.4. Uncertainty Analysis

Previous studies [19] have evaluated the theoretical uncertainty of the Moiré fringes-based alignment system using the GUM (Guide to the Expression of Uncertainty in Measurement) method [38]. However, given that we employed different alignment marks, an evaluation of uncertainty within the experimental system of this study is still necessary. This evaluation will quantify the contributions of grating quality (including grating period, surface quality, line shape, etch depth, manufacturing equipment stability, and the fabrication process) as well as external environmental factors that affect phase extraction (such as temperature variations, mechanical vibration, air turbulence, electromagnetic interference, light source stability, optics component quality, and PZT movement) to the ultimate alignment accuracy. Assuming that all uncertainties are independent of each other, these uncertainties are treated as type B uncertainties for evaluation. Each uncertainty component is determined according to a uniform distribution based on information from the instrument manufacturer’s manual and relevant references [19,39]. The detailed results of the uncertainty analysis are summarized in Table 3, with the combined uncertainty calculated using the GUM method yielding a value of 0.56 nm.

## 5. Discussion

When NIL-aligned Moiré fringes are applied in practice, the effect of noise is typically not severe. However, practical applications necessitate high efficiency. The global 2D-FFT requires less implementation time than the local 2D-WFF and 2D-FWT. Nevertheless, local phase extraction algorithms can be used if higher alignment accuracy is required. Notably, according to the experimental results, the 2D-FWT can satisfy the requirements of both real-time and high-precision NIL, enhanced to the nanometer level, which is 15.8% and 6.6% higher than that of FFT and WFF, respectively.

The actual resolution of 6.07 nm for NIL alignment applying the 2D-FWT algorithm is affected by a variety of errors, as summarized in Appendix A (Table A1). These errors mainly affect phase extraction and grating fabrication accuracy and introduce an uncertainty of 0.56 nm, and thus, their effects must be minimized to improve the experimental results. In general, the alignment accuracy is expected to increase in higher-quality experimental conditions. To improve the performance of the algorithm, future research could consider the following aspects: First, the effect of system errors such as mechanical vibrations can be reduced by employing advanced control and stabilization techniques. Second, the algorithm parameters can be optimized to balance the noise suppression and preservation of critical alignment features. Third, advanced image processing techniques can be introduced to enhance the robustness of the algorithm in complex alignment scenarios.

The accuracy observed in our experiments is slightly higher than the previously reported values [40,41], and the Std error is significantly lower, as listed in Table 4. The Std error reflects the fluctuation amplitude of multiple experimental results, serving as a measure of the stability of the experimental system, and revealing the anti-noise and anti-interference capabilities of the 2D-FWT algorithm. A smaller Std error implies consistency of experimental results and reliability of the 2D-FWT algorithm, enabling robust nanoscale lithography alignment measurements. Future work can be aimed at evaluating the performance of the algorithm in a more extensive experimental setting, including different types of imprint lithography systems and different substrate materials. These experiments can facilitate the comprehensive assessment of the generality and applicability of the algorithms for application in actual nanofabrication processes. 

We propose an accurate and fast fringe phase extraction algorithm, which not only has a wide range of promising applications in this research, but also can be useful in a variety of high-precision measurements based on the fringe phase, including, but not limited to, fringe profilometry [42], digital holography [43], and visual mechanical vibration monitoring [44]. All these applications will benefit from our research, which in turn will lead to higher accuracy and more reliable measurements. We hope that this algorithm will have a wide impact in science and engineering and promote the development and advancement of related fields.

## 6. Conclusions

In summary, we propose an improved phase extraction algorithm for wafer-mask alignment in NIL named 2D-FWT. Simulations and experiments verify that our method is more accurate and faster than traditional algorithms (2D-FFT and 2D-WFF). In addition, our algorithm outperforms the existing algorithms in various scenarios, achieving nanometer-level alignment accuracy, which is 15.8% and 6.6% higher than 2D-FFT and 2D-WFF. The 2D-FWT algorithm requires less time for parameter adjustment and is thus suitable for real-time (0.26 s) NIL. With further optimization, this high-precision algorithm can be applied in high-precision measurements based on the fringe phase, such as fringe profilometry, digital holography, and mechanical vibration monitoring.

## Figures and Tables

**Table 1:** Results of theoretical alignment measurements under ideal conditions.

SD of Noise/Gray Level	1	2	3	4	5	6	7	8	9	10
2D-FFT/nm	0.314	0.342	0.381	0.402	0.457	0.485	0.493	0.560	0.591	0.686
2D-WFT/nm	0.291	0.320	0.351	0.376	0.414	0.420	0.442	0.469	0.495	0.574
2D-FWT/nm	0.280	0.300	0.344	0.364	0.412	0.395	0.428	0.436	0.464	0.531

**Table 2:** Max error, mean error, and Std error for the misalignment values obtained by the algorithms in the original and sensitivity comparison experiments. Units: nm.

	Original Experiment			Sensitivity Comparison Experiment		
	Max Error	Mean Error	Std Error	Max Error	Mean Error	Std Error
2D-FFT	8.54	7.21	0.799	9.31	8.32	0.973
2D-WFT	7.65	6.50	0.804	8.74	7.76	1.034
2D-FWT	6.75	6.07	0.544	7.83	7.34	0.713

**Table 3:** Detailed uncertainty analysis.

$\mathrm{Component}\;u(x_{i})$	Source of Uncertainty	$\mathrm{Uncertainty}\;u(x_{i})(nm)$	$\mathrm{Sensitivity}\;c_{i}$
u(gp)	Grating period	200/√3	1 × 10^−7^
u(gq)	Grating surface quality	100/√3	1 × 10^−6^
u(gs)	Grating line shape	60/√3	1 × 10^−7^
u(gd)	Grating etching depth	80/√3	1 × 10^−6^
u(es)	Equipment stability	50/√3	1 × 10^−6^
u(gm)	Grating manufacturing process	300/√3	1 × 10^−6^
u(tv)	Temperature variation	3/√3	1 × 10^−5^
u(mm)	Mechanical vibrations	80/√3	1 × 10^−5^
u(af)	Air turbulence	5/√3	1 × 10^−6^
u(ei)	Electromagnetic interference	90/√3	1 × 10^−5^
u(ls)	Light stability	100/√3	1 × 10^−3^
u(Oq)	Optical component quality	60/√3	1 × 10^−3^
u(pz)	PZT jitter	30/√3	1 × 10^−2^
Combined uncertainty $u(x)={\left[\sum {\left(c_{i}u(x_{i})\right)}^{2}\right]}^{0.5}=0.56\;nm$			

**Table 4:** Comparison of the max error, mean error, and Std error of the misalignment measurements obtained by our proposed algorithm and previous studies. Units: nm.

Method	Max Error	Mean Error	Std Error
Ref. [40]	22.8	11.52	8.823
Ref. [41]	18.7	6.738	5.302
2D-FWT (ours)	6.75	6.07	0.544

## Data Availability

The original contributions presented in the study are included in the article, further inquiries can be directed to the corresponding authors.

## Appendix A

**Table A1:** Descriptions of experimental system error sources.

Error Source	Description
(1). Alignment mark fabrication error	Mark fabrication errors affect the Moiré fringe imaging quality. Although Moiré fringe can reduce grating etching errors, fabrication errors may lead to fringe distortion. Errors such as period deviations, etching position shifts, and shape irregularities affect the imaging and thus the alignment accuracy.
(2). Alignment signal error	When the truncate fringe length is not an integer period, the truncate error may lead to spectral leakage, thereby limiting the phase extraction accuracy. In addition, the fringe is affected by the imaging contrast, which in turn affects the phase extraction quality. The in-plane angular displacement misalignment of the two markers will produce a tilted moiré fringe and affect the radial displacement measurement, so the two alignment markers need to be leveled before the experiment.
(3). Image collection error	When collecting fringe images using CMOS cameras, spectral leakage may occur owing to the difference between the CMOS pixels and fringe periodicity. In addition, image collection is inevitably affected by dark currents and circuit noise, among other factors. These aspects may lead to phase variations and alignment errors.
(4). Phase algorithm error	In fringe analysis, phase extraction and unwrapping algorithms may induce phase errors. Specifically, the accuracy is affected by noise, image distortion, signal-to-noise ratio, and the sampling interval. Unwrapping algorithms encounter challenges in scenarios involving phase discontinuities, especially when the phase variation is large or there exist multiple discontinuities.
(5). PZT movement error	The movement error of PZT may lead to the accumulation of step experiment errors, thereby adversely affecting the alignment accuracy.
(6). Wafer and mask stress distortion	During NIL alignment, contact between the wafer and mask may lead to changes in the local stress, resulting in distortion of the alignment marks. This distortion can directly affect the imaging quality of the fringe with shape distortion.
(7). Environmental disturbances	Although we construct the experimental system on an optical platform inside an ultra-clean vibration isolation laboratory, external vibrations and airflow disturbances cannot be eliminated. These disturbances can affect the stability of the alignment marks and imaging quality of the Moiré fringe, thereby influencing the experimental results.
